# Evaluation of Different Forms of Topical Anesthesia Agents in Dental Practice

**DOI:** 10.3390/children12050610

**Published:** 2025-05-07

**Authors:** Kenan Cantekin

**Affiliations:** Department of Pediatric Dentistry, Faculty of Dentistry, Sakarya University, Sakarya 54050, Turkey; kenancantekin@sakarya.edu.tr

**Keywords:** pediatric dentistry, dental anxiety, local anesthesia

## Abstract

**Purpose:** The objective of this research was to compare the pain-reducing effects of two topical anesthetic agents, 10% atomized lidocaine spray and an EMLA, cream before needle injection applied at different time intervals using parameters of visual analog scale (VAS) score and heart rate (HR). **Methods:** The randomized split-mouth study included 30 patients (17 boys, 13 girls) aged 8.22 ± 1.8 years. The application of atomized lidocaine spray or cream was randomly used in the maxillary second premolar region. The parameters were measured prior to and following each needle insertion after being applied for 10, 30, 60, and 120 sec. Paired *t*-test and independent *t*-test were used for statistic analyses. **Results:** Compared with the first applications (10 s), atomized lidocaine and EMLA cream applications significantly decreased scores of VAS at the 30 and 120 s applications, respectively. Despite atomized lidocaine showing an early effect compared with EMLA, there were no significant differences in VAS scores between the atomized lidocaine and EMLA cream at the 60 and 120 s measurements. Although HR significantly increased at first anesthetic administration with the atomized lidocaine spray, HR significantly decreased at 30 and 120 s administrations. **Conclusions:** Atomized 10% lidocaine-based topical anesthetics significantly reduced pain more rapidly and better than EMLA from needle pricks in the buccal mucosa. Therefore, atomized lidocaine topical anesthesia could be used as a substitute for EMLA cream prior to buccal anesthetic administration. On the other hand, further comprehensive studies are required to explore the effects of several doses of atomized lidocaine in various areas of the oral cavity.

## 1. Introduction

Fear of dentists is generally due to needle injections. Topical anesthetics are most commonly used in dentistry before the application of a local anesthetic injection to mucosal surfaces. Patients suffer from two pain types during local anesthesia in the oral mucosa. One type of pain is at the insertion point of the needle, and the other is during injecting the agent. Topical anesthetics are generally used for analgesia prior to needle insertion [1,2]. Topical anesthesia is effective in the mucosa for a few millimeters (2–3 mm) and is used in pediatric dentistry for restorative dentistry, pediatric orthodontics, clamp placement, etc. [3]. Two of the most commonly used oral mucosal topical anesthetic agents worldwide are 2.5% lidocaine and 2.5% prilocaine (L/P) EMLA^®^ cream (Astra USA Inc., Westborough, MA, USA) [4]. However, since conventional topical anesthetic agents have little bio-adhesiveness to the oral mucosa, anesthetic gels often migrate from the application area, resulting in insufficient analgesia [2]. Additionally, anesthetic gels diluted by saliva may produce an unpleasant taste and discomfort for the patient [5].

Several methods for increasing the absorption of local anesthetic agents in the oral mucosa tissue have been developed and reported. Some of these methods include iontophoresis, which uses a small electric current to speed the penetration of local anesthetic agents into the gingival tissue [6,7], phonophoresis, which administers ultrasonic waves [8,9], and needleless local anesthetic systems (Injex; Pharma AG, Berlin, Germany) [10]. However, all of these methods need specialized equipment and complicated techniques, thus, they are not commonly used.

Mucosal atomization devices are devices that atomize drugs into a fine mist of particles 30–100 microns in size. First-pass metabolism is avoided; therefore, atomized nasal medications are the optimal size for rapid absorption across mucosal membranes into the bloodstream [11,12].

Mucosal atomization devices (MADs) have been used for the delivery of topical anesthetics to the naso- and oropharyngeal mucosa for approximately 15 years [13]. They are also used intranasally in pediatric emergency departments to reduce the level of anxiety, stress, and anger in patients [14]. Previous studies have shown that MADs increase the volume and distribution of delivery compared with traditional medical sprays when used prior to oro- and nasotracheal intubations [13,15,16,17]. These features may help to escalate patient compliance and refine clinical efficacy without maximizing systemic side effects. Additionally, MADs are comparatively more affordable (TRY 3 per device) and very mobile. In a recent study, MAD was used intranasally for behavior management for dental sedation [18].

In another study, intranasal midazolam was applied with MAD in a pediatric emergency dental clinic [19].

**Figure 1:**
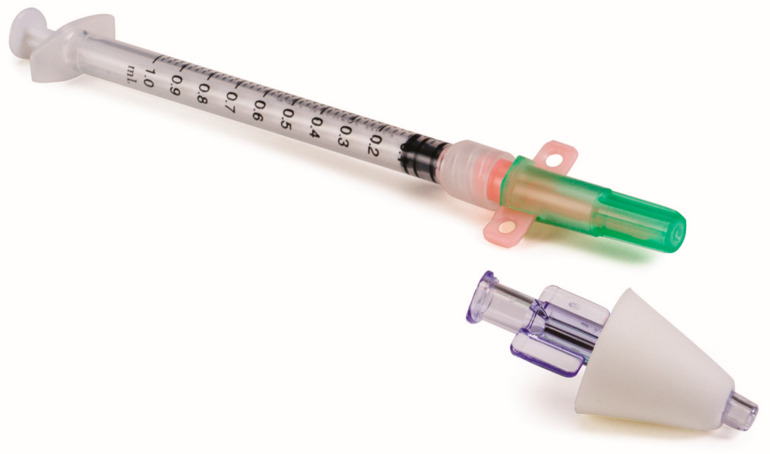
The LMA MADdy™ pediatric atomizer device that was used in the present study.

Recently, a pediatric MAD (LMA MADdy™, Teleflex Medical, New York City, NY, USA) was developed for intraoral applications. The LMA MADdy™ consists of a small atomizing tip at the end of a flexible applicator that is partially concealed by a colorful, child-friendly blowfish used to dispense topical medications to the nose, mouth, throat, hypopharynx, larynx, and trachea in a fine, gentle mist (Figure 1).

The purpose of this research was to compare the topical anesthetic effect of a 10% atomized lidocaine spray and an EMLA cream at different time intervals using both objective parameters (vital findings in pulse oximeter) and self-reported parameters (visual analog scale) prior to needle injection.

## 2. Materials and Methods

This randomized split-mouth study was managed according to the Declaration of Helsinki. The study protocol was approved by the Ethics Board of the Medical Faculty of Erciyes University, Kayseri, Turkey, and all patients gave their written informed consent to participate before inclusion in the study.

Healthy, cooperative children with a score of 4 according to the Frankl scale [20], between 7 and 10 years of age, were selected for this study among patients attending the clinic in the Department of Pediatric Dentistry, Erciyes University, Kayseri, Turkey.

Each child had at least one right and one left maxillary second primary molar with caries not requiring pulpotomy/pulpectomy treatment. A supervisor (KC) randomized and noted the application side and a single operator throughout the study performed all the procedures. The child was blinded to the type of the product being used (EMLA or MADdy). Another blinded person recorded the VAS and HR scores.

Although a single experienced clinician performed all of the anesthesia and treatment procedures, the suitability of patients, randomization of the teeth, as well as monitoring of the degree of pain, were performed by another clinician. The study was performed in a silent examination room. A power calculation indicated that 50 teeth were needed in each group to demonstrate the effect at a 91% power.

The topical anesthetics were applied on the buccal mucosal surface at the second primary molar bilaterally without letting the subjects realize which material was being applied. Atomized lidocaine was always applied to one side and EMLA cream (AstraZeneca, Södertälje, Sweden) was placed on the other side.

The sides of which material to apply was determined systematically. The supervisor researcher used an online randomizer program for the randomized application site (https://www.randomizer.org/, accessed on 11 October 2022). The buccal mucosa of the second maxillary primary molar was carefully dried, but not rubbed, with a cotton roll before the application of the topical anesthetic. The gel was applied on a cotton piece that was pressed slightly to the oral mucosa. Atomized lidocaine was applied with the use of a single-use new pediatric MAD. After the application of the agent, a 27-gauge needle was inserted in the buccal mucosa at the second primary molar region bilaterally following 10, 30, 60, and 120 s of either topical anesthetic application. The injections were always performed in the same order (i.e., first the right side and then the left side). A bleeding point from the first injection was taken as the reference point for the following injections. The tips of the injectors were not allowed to reach any contact with bone.

**Figure 2:**
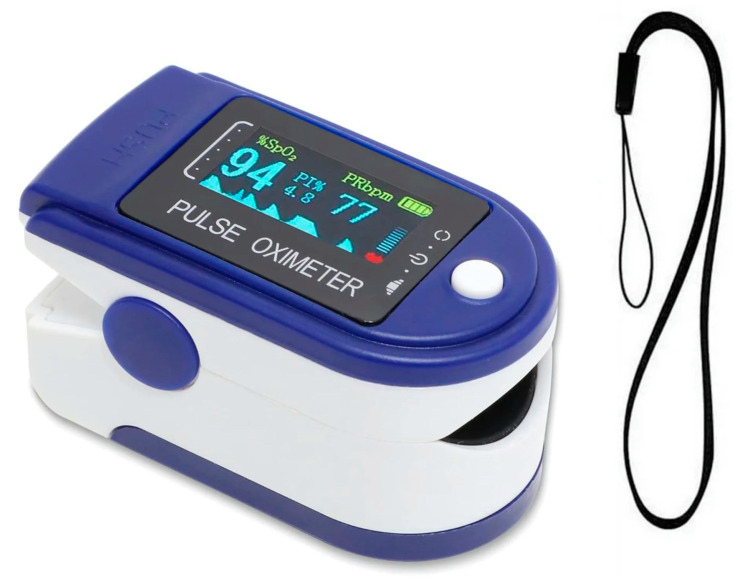
Portable pulse oximeter device that was used in present study.

To monitor the level of pain, patient responses were measured using both a modified visual analog scale [20] (VAS) and a portable pulse oximeter (CMS50-DLP model, Contec Medical Systems Co., Qinhuangdao, China) (Figure 2), which measured heart rate (HR) both before needle insertion and at needle insertion. The child was told to mark their response to pain over the VAS as explained before the injections.

The VAS ratings and heart rate scores were compared for all of the evaluated criteria using a paired sample’s *t*-test to determine if there was a statistically significant difference between the atomized lidocaine spray and the EMLA cream. The *t*-test was also used to determine if the differences in mean values between groups of different ages and genders were statistically significant. The significance level was set at *p* ˂ 0.05.

## 3. Results

The mean age of the 30 patients (17 boys, 13 girls) was 8.22 ± 1.8 years. None of the participants presented water or gag reflexes during the treatment period. The results of the VAS and heart rate (HR) scores are presented in Table 1 and Table 2. Regarding gender comparisons, there were no significant differences in VAS or HR scores (*p* > 0.05).

### 3.1. VAS Scores

VAS pain rating scores at different insertions of the needle in the buccal mucosa are shown in Table 1. Compared to the first applications (10 s), atomized lidocaine and EMLA cream applications significantly decreased scores of VAS at the applications at 30 and 120 s, respectively. Despite atomized lidocaine showing an early effect compared to EMLA, there were no significant differences in VAS scores between the atomized lidocaine and EMLA cream at the 60 and 120 s measurements.

### 3.2. Heart Rate

The mean heart rate was 108.83 (Mean) ± 11.86 (standard deviation) bpm during the test period. There were significant differences in terms of heart rates between atomized lidocaine and EMLA treatments at 30, 60, and 120 s. The mean heart rates during the study are shown in Table 2.

Although HR significantly increased at the first anesthetic administration with the atomized lidocaine spray, HR significantly decreased at the 30 and 120 s administrations.

## 4. Discussion

Children who are traumatized by dental phobia may carry this burden for their whole life. Therefore, this risk should and can be lowered with local and topical anesthetics. The pain occurring during the administration of a local anesthetic performed before a dental treatment may cause children to become dental phobic or become uncooperative for dental treatments [20]. Therefore, topical anesthetic agents are most commonly used prior to local anesthesia injections to reduce the pain from needle insertion. Although several types of topical anesthetics have been used in dentistry, EMLA cream is one of the most frequently used topical anesthetic agents by dentists [5,21,22,23,24]. On the other hand, since EMLA is not always strong enough to prevent the pain caused by needle insertion to the oral mucosa due to salivary dilution, other topical analgesics have been tested as possible alternatives to analgesic cream [5].

Atomized lidocaine has been widely used in the medical field, especially with nasogastric tube placement and several studies have examined its efficiency as an analgesic. However, for local dental anesthesia, this is the first study to use the LMA MADdy™ atomized spray, which consistently produces a fine spray of approximately 30 microns. This in vivo study has demonstrated that the application of topical anesthetics before needle insertion using a 10% atomized lidocaine was significantly more effective than EMLA in reducing pain. However, while applications of atomized lidocaine spray for 30 s were enough to prevent pain from needle insertion, EMLA cream must be used for at least 120 s before it significantly decreases pain.

Atomized lidocaine demonstrated superior properties when compared with EMLA cream. For instance, it had a rapid onset of action, it was easily applied, and it stayed in place after its application. One of the biggest disadvantages of EMLA is that it is diluted by saliva because of both its slow absorption and its cream-like nature [5]. Therefore, atomized lidocaine may be a better alternative for intraoral use.

In children, pain measurements are clearly a challenge because they are dependent on several physiological and psychological factors. Various pain measurement scales have been used to determine the level of pain in humans. Several studies have evaluated children’s self-reports of pain intensity and reported that VAS shows good sensitivity and validity for most children over six years of age [5,25,26,27]. These studies also found that self-reports, when used together with observer reports of pain, can provide a valuable indication of treatment outcomes in both clinical and research contexts [5,25,26,27]. Conversely, the regulation cardiovascular neural activity results from the combined effect of ongoing excitatory reflex interactions [28]. Under normal physiological conditions, these reflexes engage in a dynamic closed-loop interaction with rhythmic fluctuations in hemodynamics, driven by processes like respiration and vasomotor activity. Therefore, it was suggested that blood pressure and heart rate could serve as indicators of neural control, specifically reflecting the balance between sympathetic and parasympathetic influences on the cardiovascular system [25,29,30]. In the present study, VAS was used to evaluate the subjective pain and the pulse oximeter recorded objective pain since this combination of measurements has been shown to be reliable in children [31].

With regard to children’s reactions to intraoral injections, girls and boys exhibited similar levels in the present study. Concurring with our study, Ram and Peretz [32] and Allen et al. [33] reported no significant gender-specific differences in children’s reactions to intraoral injections. On the other hand, Peretz [34] observed that girls reported significantly higher pain scores compared to boys. The data were collected from self-reported questionnaires filled out by patients while waiting for their dental procedure. In the present study, children had good communication and cooperation with the operator, and it is possible that the explanation of the benefits of topical anesthesia prior to the dental procedure could have limited the pain scores.

## 5. Conclusions

Based on the results of this study, the following conclusions can be made:

1. Atomized 10% lidocaine-based topical anesthetics significantly reduced pain more rapidly and better than EMLA from needle pricks in the buccal mucosa. Therefore, atomized lidocaine topical anesthesia could be used as a substitute for EMLA cream before buccal anesthetic administration.
2. On the other hand, this is the first study that has evaluated atomized lidocaine use in dentistry. Therefore, further comprehensive studies are required to explore the effects of several doses of atomized lidocaine in various areas of the oral cavity.

## Figures and Tables

**Table 1:** The VAS scores (mean ± SD) during the applications.

	10 s	30 s	1 min	2 min
*p* (For different time)	A	B	B	B
LMA Maddy spray (mean ± SD)	1.73 ± 2.08	1.36 ± 2.35	0.90 ± 1.47	0.89 ± 1.42
*p* (For different time)	A	A	A	B
Emla cream (mean ± SD)	1.84 ± 3.01	1.81 ± 2.65	1.74 ± 2.05	1.50 ± 2.14
*p* (Between topical analgesics in same time)	NS (*p* = 0.86)	NS (*p* = 0.48)	* (*p* = 0.04)	* (*p* = 0.01)

Same letters indicated no significant difference. NS, there was no significant difference. *, there was a significant difference.

**Table 2:** Heart rate and oxygen saturation during atomized lidocaine spray and benzocaine cream application.

Heart Rate					
	Before application	10 s	30 s	1 min	2 min
LMA Maddy spray	105.10 ± 12.53 A	117.91 ± 14.83 B	110.50 ± 11.96 A	107.43 ± 10.91 A	106.73 ± 10.61 A
Emla cream	103.97 ± 12.59 A	112.36 ± 12.95 B	112.85 ± 11.65 B	110.44 ± 11.61 B	107.53 ± 15.72 B
*p*	NS	NS	*	*	*
Oxygen saturation					
	Before application	10 s	30 s	60 s	2 min
LMA Maddy spray	96.7 ± 1.94 A	96.5 ± 2.04 B	97.7 ± 1.55 A	97.4 ± 1.33 A	97.1 ± 1.28 A
Emla cream	97.36 ± 2.32 A	95.3 ± 2.23 B	95.5 ± 2.28 B	96.12 ± 2.88 B	97.56 ± 2.01 B
*p*	NS	NS	*	*	*

*, there was significant difference; same letters indicated no significant difference. NS, there was no significant difference.

## Data Availability

The original contributions presented in the study are included in the article, further inquiries can be directed to the corresponding author/s.
